# Using normalisation process theory to understand implementation of effective early-onset type 2 diabetes treatment and care within England: a qualitative study

**DOI:** 10.1186/s12913-025-12616-w

**Published:** 2025-03-24

**Authors:** Radhika Chauhan, Melanie J Davies, Carl May, Shivani Misra, Jack A. Sargeant, Mike Skarlatos, Jane Speight, Emma G. Wilmot, Caroline Wilson, Michelle Hadjiconstantinou

**Affiliations:** 1 Division of Psychology, School of Applied Social Sciences, Faculty of Health and Life Sciences, De Montfort University, Leicester, UK; 2 Diabetes Research Centre, University of Leicester, Leicester, UK; 3 Leicester Diabetes Centre, University Leicester Hospitals Trust, Leicester, UK; 4 NIHR Leicester Biomedical Research Centre, University Hospitals of Leicester NHS Trust and University of Leicester, Leicester, UK; 5 Faculty of Public Health and Policy, London School of Hygiene and Tropical Medicine, London, UK; 6 Division of Metabolism, Digestion and Reproduction, Imperial College London, London, UK; 7 Department of Diabetes and Endocrinology, Imperial College Healthcare NHS Trust, London, UK; 8 Australian Centre for Behavioural Research in Diabetes, Diabetes Victoria, Melbourne, Australia; 9 Diabetes Department, University Hospitals of Derby and Burton NHS Foundation Trust, Derby, UK; 10 University of Nottingham, Nottingham, UK

**Keywords:** People living with early-onset type 2 diabetes, Healthcare professionals, NPT, Implementation, Specialist care

## Abstract

**Background:**

Despite increasing prevalence, early-onset type 2 diabetes (EOT2D) has received little clinical and qualitative research attention within England. This qualitative study aimed to explore and understand the unmet needs of people living with early-onset type 2 diabetes (PEOT2D) and their diabetes care within England.

**Methods:**

Using semi-structured interviews, data was collected, transcribed and analysed from 25 PEOT2D and 25 healthcare professionals (HCPs). Taking an abductive approach, data for both cohorts were analysed and interpreted according to four constructs of Normalisation Process Theory (NPT): *coherence* (sense-making), *cognitive participation* (engagement), *collective action* (enactment) and *reflexive monitoring* (formal and informal appraisal).

**Results:**

Our findings revealed several unmet needs in current treatment and care for PEOT2D. The main unmet need was access to specialist care. Having GP (general practitioner) practices as their main caregivers presented a significant barrier to this population successfully carrying out their diabetes self-care. HCPs in specialist roles expressed similar views and were keen to see PEOT2D receive access to holistic and specialist care via a multidisciplinary team. Data interpretation according to the four constructs of NPT found that implementation of this approach would involve fostering an environment of support that allowed HCPs across the primary and secondary interface to do the following: (1) provide consultations incorporating person-centred care, shared decision-making, and non-judgemental and non-stigmatising behaviours and (2) work in an integrated and synchronous manner using streamlined referrals, interprofessional collaborations and team-based learning. Provision of tailored financial, human (additional staffing) and learning resources was found to be integral to allow creation of tailored multidisciplinary teams, and individual and collective skill enhancement of both specialist and primary care providers.

**Conclusion:**

Although both PEOT2D and specialist care providers are keen for young adults with EOT2D to receive access to specialist and holistic care, there are several resource barriers that must be addressed to allow implementation of their desired approach to treatment and care. Further qualitative research with primary care providers (for example, GPs and practice nurses) involved in EOT2D care is needed to understand if (and how) their views and experiences differ from those providing specialist care.

**Supplementary Information:**

The online version contains supplementary material available at 10.1186/s12913-025-12616-w.

## Background

Previously considered a health condition of mid to late adulthood, type 2 diabetes (T2D) has become increasingly common in children, adolescents, and young adults (aged 16–39 years) [1–4]. Findings published by the National Diabetes Audit (2021-22) revealed there are 139,355 individuals under 40 years living with T2D in England. The majority of these (138,420) are young individuals aged 16 to 39 years [5]. The onset of T2D in young adulthood (16–39 years) is more commonly known as early-onset type 2 diabetes (EOT2D).

Compared to their older counterparts (40 years and above), people with early-onset type 2 diabetes (PEOT2D) are at greater risk of developing multiple diabetes-related microvascular (retinopathy and neuropathy) and macrovascular (coronary artery disease and stroke) complications [3, 4, 6]. Premature development of these complications also places these young adults at greater risk for premature mortality [4, 6]. Findings published by Rhodes et al. [7] reported a 15-year reduction in the average remaining life expectancy (RLE) for PEOT2D, when compared to the anticipated RLE for 20 year olds without diabetes in the United States [7]. Findings also show PEOT2D to be more likely to experience psychological complications such as depression, anxiety, diabetes-related distress and low self-compassion [8, 9].

Despite the increasing prevalence and poor outcomes, treatment and care for EOT2D is primarily driven by research evidence extrapolated from older adults (40 years and above) [10–12]. There is an urgent need for tailored care strategies to effectively support this high-risk group [12–14]. Unlike their older counterparts, PEOT2D are often facing major life transitions such as independent living, study, work/early careers, geographical relocation, establishment of new friendships and romantic relationships, family planning and/or raising children. These events can take precedence over diabetes care and contribute to the physical, psychological, and emotional challenges faced by PEOT2D [1, 8, 10].

Understanding the differing needs and priorities of PEOT2D is essential to support the development of effective treatment and care for these individuals [15]. However, to date, PEOT2D remain highly underrepresented in clinical and qualitative research [8, 10, 15]. Therefore, there is limited evidence-based knowledge on their unique needs and preferences to inform the design of tailored care services [10, 11, 15].

Current research into the unmet of needs PEOT2D stems from two Australian studies, a quantitative study by Browne et al. and a qualitative study by Savage et al. respectively [15, 16]. Findings published by Browne et al. showed that 68% of young people with T2D perceived their healthcare needs differed to their older counterparts and there was a need for targeted and age-specific information and services (62%) [15]. Similarly, Savage et al. revealed themes pertaining to the need for age-specific educational and informational resources for young people with T2D, according to their content and delivery format preferences [16]. Recognising the growing prevalence of EOT2D within England [5], this qualitative study aimed to fill a demonstratable knowledge gap by exploring and understanding the unmet needs of these individuals and their EOT2D care. To fulfil the research aim and ensure an in-depth exploration of the unmet needs of EOT2D treatment and care this study proposed the following objectives:

- To understand the current challenges of living with and managing EOT2D from the perspectives of young adults with the condition.
- To understand healthcare professionals’ views and perspectives regarding current T2D treatment and care.
- To gather and identify potential improvements in EOT2D treatment and care from both a personal (PEOT2D) and clinical (healthcare professional) perspective.

## Methods

Interviews were designed to explore the challenges of living with EOT2D and gaps in T2D care planning for these young adults. Research Ethics Committee (REC) favourable opinion and HRA approval was obtained from the East Midlands, Nottingham 1 REC (22/EM/0014).

### Participant recruitment and sampling

#### People with early-onset type 2 diabetes

PEOT2D aged 16 to 40 years (inclusive) were eligible to participate in this study; with those aged 16 and 17 years covered by the Gillick Competence Act. Recruitment included 11 GP (general practitioner) practices with the support of the National Institute for Health and Care Research (NIHR) East Midlands Clinical Research Network (CRN), and from hospital specialist services including: the University Hospitals of Leicester NHS Trust (UHL), Nottingham University Hospitals NHS Trust, University Hospitals of Derby and Burton (UHDB) and Imperial College Healthcare NHS Trust. Additional recruitment avenues included North-West London CRN, advertising on social media and ‘word of mouth’.

#### Healthcare professionals

HCPs aged 18 to 75 years (inclusive) who had been and/or were currently in contact with people with T2D were eligible to participate in this study. Efforts were made to recruit a diverse range of HCPs using the following avenues: GP practices and hospital specialist services, social media advertising, poster distribution at national and local training events and meetings, and existing interprofessional networks. For further details about participant recruitment see Table 1.

All potential participants that expressed an interest in participating were provided with study invitation packs. Two packs were created: one for the PEOT2D and one for the HCPs. Reply slips entailing sociodemographic questions were enclosed in both invitations packs. All study documents received input from patient and public involvement members.

**Table 1:** Recruitment source for HCPs and PEOT2D

Recruitment Sources	HCPs	Young adults with EOT2D
Primary Care (North West London CRN)	0	2
Primary Care (East Midlands CRN)	1	3
Secondary Care (UHL)	1	9
Secondary Care (UHDB)	2	9
Centre for Ethnic Health Research	n/a	1
Social Media	3	0
Invitation to eligible adults who consented to be contacted for future research	n/a	1
HCPs and Allied Health Professionals Networks (i.e. DSN, Dietetic)	7	n/a
Existing Networks with Diabetes Clinics	11	n/a

### Data collection

Two semi-structured topic guides were developed for this study: one for the PEOT2D and one for the HCPs (see Supplementary Materials 1 and 2). Questions informed by the Capability-Opportunity-Motivation-Behaviour (COM-B) model [17, 18] were incorporated into both topic guides to facilitate exploration of individual capacity, opportunity, and motivational factors that may impact daily self-management behaviours amongst PEOT2D. A theory of implementation, Normalisation Process Theory (NPT) [19], also informed development of both topic guides to elicit data that allowed identification of factors that promote and inhibit implementation of T2D care at an individual and collective level from both an individual (PEOT2D) and healthcare professional (HCP) perspective.

Interviews were conducted between July 2022 and May 2023 by a qualitative research assistant with a background in Psychological Well-being (RC). A Behavioural Scientist and experienced qualitative researcher (MH) co-facilitated the first interview with both participant cohorts and led reflective discussion about the topic guides and emerging interview data. There were no pre-existing relationships between the participants and interviewer (RC) prior to commencement of the study. Before interviews, participants were informed about the interviewer’s goal of the research i.e. understand more about current EOT2D treatment and care from a personal (PEOT2D) and clinical (HCP) perspective, and suggestions for improvements. Given the interviewer did not have personal lived experience of EOT2D and nor had they worked as a healthcare provider, they were aware of their outsider status and the potential misinterpretation of participants views and perspectives of EOT2D that could result from this. To mitigate against this, the interviewer made sure to familiarise themself with the existing literature in the topic area and approach all interviews with empathy and an open mind. Reflexivity was also key, with the interviewer (RC) writing field notes and personal reflections after the completion of each interview. All interviews were audio recorded and took place via telephone, online (via Microsoft Teams) or face-to-face depending on participant preference and covid-19 guidelines. Interviews with PEOT2D ranged between 10 and 81 min whilst interviews with HCPs ranged between 52 and 73 min. Informed consent was obtained from all participants prior to their participation in the interview.

### Data analysis

All audio files were transcribed verbatim by a professional transcription service. Anonymised transcripts were subsequently uploaded onto qualitative indexing software (NVivo (v12.0)), for coding and analysis.

Taking an abductive approach [20], data for both PEOT2D and HCPs were first analysed to develop an initial set of codes that reflected the interview data. Prior to doing so, all transcripts were read and reread to ensure familiarisation with the data. Transcripts were coded independently by RC, supported by regular meetings with MH to discuss coding and ensure coding agreement.

Aligning with the abductive approach, initial codes and data for PEOT2D and HCPs were re-reviewed to deem their suitability for interpretation according to constructs of NPT. This purposeful action allowed us to thoroughly consider the implementation potential for the desired EOT2D treatment and care that emerged from our dataset. Thereby, aligning with previous research that has emphasised the need to eliminate gaps between healthcare research and real-world implementation [21]. Organising data analysis in this way ensured that the qualitative study remained open to analytic results that was neither deliberately specified or predicted by theory, nor driven by the interviewer’s (RC) bias or assumptions. After in-depth discussion, RC and MH agreed for codes from both participant cohorts to be mapped onto NPT using the NPT coding manual [19].

### Normalisation process theory

NPT is an implementation theory focused on understanding the *work* that individuals and groups of people do, to make practices (often complex interventions) routine in everyday life, usually in the context of healthcare delivery [19, 22, 23]. The theory identifies, describes, and explains four key mechanisms (Coherence, Cognitive participation, Collective action and Reflexive monitoring) for implementing, embedding, and integrating practices into social contexts. Coherence explores the sense-making or understanding of a practice. Cognitive participation refers to engaging with a practice. Collective action refers the enactment of a practice and reflexive monitoring refers to the formal and informal appraisal of a practice [19, 23, 24].

Cognisant of the predominant use of NPT as an implementation evaluation tool [25], our analytic approach involved adapting use of the NPT coding manual [19] and existing relevant literature [24, 26] to create distinct NPT-informed coding frameworks for both PEOT2D (see Table 2) and HCPs (see Table 3). Development of the frameworks and mapping of codes according to NPT constructs was an iterative process, with RC and MH meeting on a regular basis to discuss and refine the framework and data interpretation as needed. As theory developer, CM, also provided expertise and guidance on the analytic approach. An expert in health behaviour change, JS, also provided input and guidance into data analysis.

Recognising that our dataset captured two distinct perspectives, the current lived experiences of PEOT2D and HCP recommendations for future EOT2D treatment and care, we developed tailored coding frameworks for each cohort accordingly. Despite the NPT coding manual outlining 12 primary constructs broken down according to the *Context-Mechanism-Outcome* framework [19], we chose to focus on the constructs (and subconstructs) associated with *Mechanisms* (as outlined above). This is because the Mechanism constructs are central to NPT [21]. They provided the foundation to establish both the coding manual and health-related NPT research [19, 22]. Nevertheless, to ensure comprehensive use of the manual and to fully consider the implementation potential of our data, we also attempted to map our codes onto the constructs associated with Contexts (the social structures and relations that make up and effect the implementation environment) and Outcomes (changes/effects due to implementation). However, this proved difficult as they were more aligned with data emerging from implementation evaluation studies [19]; therefore, we excluded these from both coding frameworks.

**Table 2:** NPT coding framework for PEOT2D (adapted from Gallacher et al. [26], May et al. [19])

NPT Constructs			
**Coherence (Sense-making)**: Understanding the prospect of living with EOT2D, what this means and how the condition will be managed	**Cognitive participation (Engagement)**: Investing personal and interpersonal commitment to living with EOT2D and its self-management	**Collective action (Enactment)**: Investing effort and resources in self-management, carrying out tasks and living with EOT2D	**Reflexive monitoring (Formal and informal appraisal)**: Retroactively reflecting on the effects of EOT2D treatment and management, and deciding whether to modify plans
**Differentiation**: Understanding and distinguishing aspects of EOT2D tests, treatments and the roles of different HCPs	**Enrolment**: Engaging with friends, family, and HCPs with regards to EOT2D and its management to allow them to provide support	**Interactional workability**: Taking medication, enacting lifestyle changes, attending appointments, enduring symptoms and medication side effects	**Systematisation**: Developing strategies to stay up to date with newly available EOT2D treatment and care
**Individual specification**: Achieving a subjective understanding of EOT2D and its management, through self-learning and lived experience	**Initiation**: Using organisational skills to arrange personal contribution to management of EOT2D (e.g., arranging prescriptions and transportation to appointments)	**Skill set workability**: Setting a routine to cope with symptoms, condition exacerbations and emergency situations	**Individual appraisal**: Evaluating individually whether to continue with, or alter current EOT2D treatment and management plan
**Communal specification**: Obtaining information about EOT2D and its management with the help and support of others (e.g., friends, family and HCPs	**Activation**: Arranging help (e.g., logistical, administrative or professional) from HCPs, friends or family	**Relational integration**: Developing strong relationships with and confidence in HCPs and their interaction with each other; overcoming barriers in accessing EOT2D treatment and care	**Communal appraisal**: Discussing or altering current EOT2D treatment and management plan already initiated in discussion with HCPs, friends and family
**Internalisation**: Associating personalised experience with EOT2D and its treatment, understanding implications and knowing when to seek help	**Legitimation**: Seeking reassurance from others about suitability of EOT2D treatment and management plan	**Contextual integration**: Ensuring availability of right financial and social resources and integrating EOT2D into social circumstances	**Reconfiguration**: Altering EOT2D self-management routine when required for example, medication regimens or appointments to fit around daily activities or other personal arrangements

**Table 3:** NPT coding framework for HCPs. (adapted from Gallacher et al. [26], May et al. [19])

NPT Constructs			
**Coherence (Sense-making)**: Understanding the possibility of a different form of EOT2D treatment and care, what this means and how it will take place	**Cognitive participation (Engagement)**: Investing personal and interpersonal commitment to engage with desired EOT2D treatment and care	**Collective action (Enactment)**: Investing effort and resources to carry out desired EOT2D treatment and care	**Reflexive monitoring (Formal and informal appraisal)**: Retroactively reflecting on the desired EOT2D treatment and care and deciding whether to modify it
**Differentiation**: Distinguishing desired EOT2D treatment and care from existing practices	**Enrolment**: Engaging with other HCPs and key individuals to enable desired EOT2D treatment and care to take place	**Interactional workability**: What work will HCPs have to do to implement the desired EOT2D treatment and care?	**Systematisation**: How will HCPs and other key individuals’ gain access to information about the effectiveness and usefulness of the implemented EOT2D treatment and care?
**Individual specification**: Achieving subjective understanding of desired EOT2D treatment and care, and the work required to implement this on an individual level	**Initiation**: Recognising key individuals needed to make desired EOT2D treatment and care happen	**Skill set workability**: What skills and knowledge will HCPs require to implement the desired EOT2D treatment and care?	**Individual appraisal**: How are HCPs and other key individuals likely to perceive the effectiveness and usefulness of the desired EOT2D treatment and care on an individual level?
**Communal specification**: Achieving subjective understanding of desired EOT2D treatment and care, and the work required to implement this on a collective level	**Activation**: How will HCPs and other key individuals work together to ensure that the desired EOT2D treatment and care continues to take place?	**Relational integration**: How will the desired EOT2D treatment and care effect the confidence that HCPs and other key individuals have in each other?	**Communal appraisal**: How are HCPs and other key individuals likely to perceive the effectiveness and usefulness of the desired EOT2D treatment and care on a collective level?
**Internalisation**: Understanding the potential value/benefits of desired EOT2D treatment and care	**Legitimation**: How will HCPs agree that the desired EOT2D treatment and care is suitable and should be integrated into their existing practices?	**Contextual integration**: What resources are required to implement the desired EOT2D treatment and care into existing settings/organisations?	**Reconfiguration**: How will HCPs and other key individuals modify their existing practices following implementation of the desired EOT2D treatment and care?

## Results

### Sample characteristics

Fifty individual interviews were conducted with PEOT2D (*n* = 25 interviews) (see Table 4) and healthcare professionals (HCPs) (*n* = 25 interviews) (see Table 5).

### People with early-onset T2D

The vast majority of PEOT2D that took part in this study were female (*n* = 19, 76%), aged between 25 and 39 years (*n* = 22, 88%), had been diagnosed with T2D in the last 3 to 10 years (*n* = 15, 60%) and had at least one comorbidity (*n* = 17, 68%). Despite the ethnic diversity of the recruitment areas, most participants were from a white background (*n* = 16, 64%). Further PEOT2D characteristics can be found in Table 4.

**Table 4:** Sociodemographic characteristics of PEOT2D

	**Total (** ***n*** **= 25)**	
	**n**	**%**
**Gender**		
Male	6	24
Female	19	76
Non-Binary	0	0
Not listed	0	0
Prefer not to say	0	0
**Age**		
16–24 years	1	4
25–39 years	22	88
40 years	2	8
**Ethnicity**		
Asian or Asian British	9	36
Black or Black British	0	0
White	16	64
Mixed or multiple ethnic group	0	0
Another ethnic group	0	0
Other	0	0
**Length of diabetes diagnosis**		
Less than 6 months	3	12
Between 1–3 years	6	24
Between 3–10 years	15	60
10+	1	4
**Education**		
Secondary School	4	16
Sixth Form/College	6	24
Undergraduate Degree	10	40
Postgraduate Degree	3	12
Prefer not to say	0	0
Missing	2	8
**Confidence using Internet Resources**		
Very Confident	17	68
Confident	6	24
Slightly Confident	1	4
Not Confident	0	0
Missing	1	4
**Number of Long-term Health Conditions**		
1	7	28
2	6	24
3	5	20
4 or more	6	24
Missing	1	4

### Healthcare professionals

HCPs were successfully recruited using social media advertising (*n* = 1) and existing interprofessional networks (*n* = 24). The majority of HCPs that participated in this study were female (*n* = 17, 68%), aged between 40 and 59 years (*n* = 16, 66.7%), from a white background (*n* = 18, 72%) and working as specialist care providers (SCPs) (*n* = 24, 96%). Despite efforts to recruit HCPs across various disciplines, only one GP was interviewed. Further HCP characteristics are displayed in Table 5.

**Table 5:** Sociodemographic characteristics of HCPs

	**Total (** ***n*** **= 25)**	
	**n**	**%**
**Gender**		
Male	8	32
Female	17	68
Non-Binary	0	0
Not listed	0	0
Prefer not to say	0	0
**Age**		
18–24 years	0	0
25–39 years	8	32
40–59 years	16	64
60–75 years	0	0
Missing	1	4
**Ethnicity**		
Asian or Asian British	4	16
Black or Black British	2	8
White	18	72
Mixed or multiple ethnic group	0	0
Another ethnic group	0	0
Other	1	4
**Role in type 2 diabetes care** ^*^		
Dietitian	5	19.2
Diabetes Specialist Nurse	9	34.6
General Practitioner	1	3.8
Diabetes Consultant	6	23.1
Healthcare Assistant	0	0
Other (i.e., Registrar, SMES^**^ educators)	4	15.4
Missing	1	3.8
**Years of Experience in Role**		
6–12 months	0	0
1–5 years	6	24
5–10 years	11	44
10+ years	7	28
Missing	1	4
**Years of Experience Treating Young Adults with Type 2 Diabetes**		
None	0	0
Less than 6 months	0	0
6–12 months	0	0
1–5 years	5	20
5–10 years	10	40
10+ years	9	36
Missing	1	4
**Type of Healthcare Institution** ^*^		
Primary	7	22.6
Secondary	18	58.1
Community	5	16.1
Missing	1	3.2

^*^Selected more than one answer

^**^Self-management education and support

In accordance with our NPT-informed coding framework for PEOT2D, codes mapped onto the four *Mechanism* constructs provided a detailed insight into the personal and healthcare-related challenges experienced by PEOT2D. Below, we summarise key findings pertaining to each construct.

### Coherence: understanding EOT2D and its treatment and management

Making sense of EOT2D and the various aspects of its treatment and management proved difficult on an individual and collective level for PEOT2D. Processing and accepting the diagnosis was a key stepping stone to this population engaging with their diabetes treatment and self-management.

“I’ve finally accepted it. I would say it’s only been within the last year or two I’ve accepted it and I think I’m coming into my thirteenth or fourteenth year. And with that came not treating my diabetes, avoiding it.”

(P19, Female, 25–39 years)

On a collective level, various aspects of asynchronous care exacerbated the difficulties PEOT2D experienced in learning about and understanding different aspects of the tests and treatments relating to their condition. For example, seeing different HCPs meant PEOT2D received conflicting information about their medication and treatment which often attributed to unrealistic expectations about their remission prospects. Furthermore, navigating disjointed follow-ups and lack of access to diabetes specialists meant young adults with EOT2D were left to undertake diabetes self-management research alone. Consequently, PEOT2D were often dealing with misinformation and trial and error when it came it to managing their condition.

“I had to kind of do my own research kind of thing, which does take its toll because you’re doing so many hours of research, you don’t know what information is true because you’re not a specialist. I don’t know anything about diabetes.”

(P01, Male, 25–39 years)

### Cognitive participation: engaging with EOT2D treatment and management

Data coded to cognitive participation revealed low levels of engagement with friends, family and HCPs amongst PEOT2D meaning they lacked the personal and professional support needed to effectively manage their condition. The lack of engagement with friends and family often stemmed from PEOT2D withholding their diagnosis from these individuals due to concerns about their reactions for example, receiving unwanted sympathy or being told how to manage their condition. Similarly, cultural perceptions and beliefs around T2D also attributed to these young adults struggling to seek support from family members when it came to managing their condition.

“My mum and dad still don’t know how to support me. They always go, what do I do, what do I do? They’re still lecturing me about diabetes. I’m like, it’s already happened now, but I end up not wanting to talk about it with them, but that’s because no one ever taught them how to talk about it, and you imagine there’s families that don’t even have that dialogue around language, culture.”

(P09, Female, 25–39 years)

The lack of access to SCPs also made engaging with HCPs difficult for PEOT2D. Having GP practices as the first point of contact for their diabetes treatment and care posed several challenges for these young adults, often in the form of poor patient-healthcare professional interactions. GP practices were perceived to lack the level of investment, care and expertise that was desired by PEOT2D.

“…because I’m type 2, primary care is given to the doctors, not a hospital, but yet I’m under the treatment that really should be under the hospital. So I’m on this bridge that nobody seems to really want to pick me up, apart from the fact of doctors want me for a health check because they want their pay cheques to do my diabetes health check. They’re not really bothered about it…”.

(P19, Female, 25–39 years)

Arranging their own contribution and engagement with diabetes-related healthcare tasks was found to be burdensome and frustrating for PEOT2D as they had to consider a multitude of physical barriers (location, time and transport) due to the demands of employment and/or parenthood. Hence, highlighting the need for provision of age-accommodating diabetes treatment and care for this priority population.

### Collective action: executing EOT2D treatment and management

The management of daily life demands such as parenting and employment made enactment of diabetes self-management behaviours much more complex for PEOT2D. Participants frequently spoke about modifying medication regimens to avoid dealing with side effects during work commutes. Enacting lifestyle changes and monitoring blood sugars also presented similar challenges with childcare and work responsibilities taking priority over self-management.

“I work in sales so I’m on the phone a lot. But that means that I can’t really just get up and go for a walk for– I can’t really do that. I have a half an hour lunch and that’s for me to literally make my food and eat it. So not enough time to go outside and do something. When you work in a job like that, everything has to be after five o’clock or before nine o’clock and it’s hard, it’s really hard to juggle everything.”

(P15, Female, 16–24 years)

Dealing with psychological, physiological and neurological comorbidities created further complexities in carrying out diabetes self-management. Therefore, it was unsurprising that many of these individuals were keen to have access to flash glucose monitors (Freestyle Libre) and continuous glucose monitors (CGM). These devices had potential to make integration and enactment of daily self-management behaviours a less emotionally and physically burdensome task.

In addition to their personal challenges, PEOT2D also identified a range of barriers to accessing treatment and care that again created difficulties in enacting healthcare behaviours and tasks. The most frequently mentioned barriers related to the various aspects of asynchronous care at the primary-secondary interface (for example, the total lack of and/or incomplete provision of patient information between different HCPs and/or electronic health record systems) and poor patient-healthcare professional interactions. Negative interactions covered a span of unfavourable communication and consultation behaviours for example, one-sided conversations, insufficient information provision and the use of judgemental and stigmatising language.

“…the attitude from one doctor I got when I rang my GP surgery for a sicknote because I wasn’t ready to go back to work was ‘well basically you’ve done this to yourself, you’ve sat on the sofa and filled your face, that’s why you’re in this position’.”

(P21, Female, 25–39 years)

PEOT2D were keen to see both barriers addressed with particular emphasis given to the need for improved HCP communication and consultation skills. Examples of preferred behaviours included the use of shared decision-making, treatment transparency and autonomy, and provision of kind and compassionate care.

### Reflexive monitoring: appraising EOT2D treatment

Asynchronous care also made it difficult for PEOT2D to assess and appraise their management plans individually and collectively. Irregular blood and pathology testing, and disjointed follow-ups meant this underrepresented group continued to follow suggested management plans for prolonged periods of time with little awareness of its suitability.

“He was like, right, you need to go on gliclazide, you know, oh, my God, your blood sugars are horrific. So, anyway, went on that, and then I was left on it for two years, because I’m not very good at taking medication, I didn’t take it that much. And then, a doctor was like, another doctor was like, oh, my God, why are you on this? I was like… He goes, you shouldn’t be on 100 milligrams of gliclazide for two years.”

(P09, Female, 25–39 years)

Furthermore, the lack of treatment autonomy and dismissive behaviour shown by HCPs made it difficult for these individuals to raise any concerns they had about their treatment; particularly when receiving care at a GP practice. PEOT2D emphasised the need for a reformed care pathway that provided them with immediate access to SCPs. This was seen as key to addressing many of the existing barriers in their treatment and care.

“If the GP surgery can’t accommodate, then why aren’t they being referred straight over for a diabetic nurse, who sees these people every day, treats them like people, and can actually have a conversation about everything and medicines and any concerns they have…”

(P08, Female, 25–39 years)

Aligning with the current lived experiences of PEOT2D, mapping of the HCP codes according to the *Mechanism* constructs of NPT also revealed the need for a different approach to future EOT2D treatment and care. Specifically, one that transferred primary caregiving responsibilities to specialists. Findings pertaining to each construct also highlighted a multitude of barriers and facilitators to implementation which, if addressed, had the potential improve the future lived experiences of PEOT2D. We discuss these findings in further detail below.

### Coherence: understanding the need for a different approach to EOT2D treatment and care

SCPs were conscious of the responsibilities and workloads of GPs, and accordingly expressed the need to provide PEOT2D with access to specialist care. Specialists felt they would be better equipped to provide the treatment and care desired by this priority population. They identified provision of holistic and personalised care via a multidisciplinary team (MDT) as the ideal standard for PEOT2D; thus, making it clearly distinct from existing care where GP practices were often the sole caregivers.

“…a GP is seeing everything and having to deal with everything. They’re not going to have the time to sit and think would they benefit from this, that and the other, so I think making sure that they do come under specialist services as opposed to being sat in GP land for fifteen years and now they’ve got complications is the key thing really.”

(HCP06, Female, 40–59 years)

SCPs felt implementing the above approach had potential to elicit multifaceted benefits for PEOT2D including greater consultation time, provision of early aggressive intervention and individualised advice, and overall improved engagement with treatment and care.

Despite understanding the need for their suggested approach, specialists were cognisant of many barriers that could hinder implementation. These included organisational barriers such as HCP roles and expectations, staff shortages and workload, appointment back logs, and the confines of existing clinical practice and guidance. Medical and financial resource barriers were also identified for example, limited pharmaceutical therapies and funding limitations.

“I think we spend so long being told that we don’t have any money for anything and it sort of cuts you off thinking that you can do anything outside the box of where you are.”

(HCP15, Female, 40–59 years)

### Cognitive participation: engaging with the different approach to EOT2D treatment and care

SCPs were aware of the high morbidity and mortality risk amongst PEOT2D and therefore, were significantly invested in engaging with and integrating their desired approach to treatment and care. However, they emphasised the need for EOT2D risk awareness and education across GP practices to facilitate the same level of engagement from primary care services. Specialists felt this would be beneficial to encourage EOT2D referrals onto specialist services.

“…the GPs wouldn’t think oh I’ve got somebody that’s 32 that’s been diagnosed with type 2, I’m going to refer. They wouldn’t, they would just stick them on metformin and go down the NICE guidance. So yeah, I think maybe a push towards educating the GPs and other health care professionals about how important it is to get these patients on board and prevent complications.”

(HCP06, Female, 40–59 years)

Similar to PEOT2D, the HCPs also recognised many shortcomings in continuity of care that could deter implementation of their desired approach to EOT2D treatment. Drawing on their experiences of type 1 diabetes, specialists suggested the need for an accessible ‘point of contact’ as a possible solution to addressing continuity issues.

“… I think there are basics about how you set it up, how do you contact people, how do you follow people up. I think sometimes it is simply relationships, so I think in the evidence for Type 1 diabetes, when people come across on the children’s one of the factors is having a coordinator or a clinician who is like their contact.”

(HCP04, Male)

There was consensus that promoting communication and collaboration across the primary-secondary interface was key to ensure engagement with the desired treatment and care on an ongoing basis. Examples of this included opportunities for interprofessional collaboration and team-based learning to promote shared ethos and values regarding overall EOT2D treatment and care.

### Collective action: executing the different approach to EOT2D treatment and care

Enacting the desired EOT2D treatment and care required a shift in the way specialist and primary care providers (PCPs) worked on an individual and collective level. As such, there was substantial overlap in data coded to cognitive participation (engagement) and collective action (enactment). On an individual level, it was highlighted that all HCPs across both primary and secondary care would have to adopt a holistic and person-centred approach to consultations rather than the more commonly used clinical model. Introducing this change would ensure that HCPs were being considerate of the many complexities (for example daily life demands and comorbidities) that impacted self-management behaviours in PEOT2D.

“…you need to have a different approach which is actually founded on the psychology of adolescence as opposed to the biomedical model. You know this is not a middle-aged person who shops at Waitrose who’s gonna take the medicine exactly when you prescribe them. This is a young person whose life is in flux. And I think a lot of physicians struggle with that…”

(HCP23, Male, 25–39 years)

Successfully implementing the above change would require increased funding for the training and upskilling of HCPs in a range of communication and consultation skills; with particular emphasis given to removal of judgemental and stigmatising behaviours.

“…they’ve got to cope with blame and shame. And there is stigma and there is attitudes, the public and the media, but healthcare professionals’ attitude is pretty rotten as well. And then, so actually, so that needs to be looked at”

(HCP02, Female, 40–59 years)

Recognising that provision of holistic and personalised care would involve them dealing with concerns and queries outside their remit and roles, SCPs also identified the need for access to trustworthy and tailored signposting materials as well as, access to a range of qualified specialists including psychologists.

“…I’m not a trained psychologist. And therefore, you know, we need and psychological care is present. We do have some. We’re very lucky in [de-identified place 1], but gosh, there is a need for more. So I think that’s a real unmet need is psychological support.”

(HCP23, Male, 25–39 years)

On a collective level, again, coordination and collaboration across the primary-secondary interface was key to carrying out the desired EOT2D treatment and care. For those working in primary care this would involve a significant shift from their existing practices as they would be navigating the implementation of a revised referral and care pathway in which, they were no longer the main caregivers for PEOT2D.

### Reflexive monitoring: appraising the different approach to EOT2D treatment and care

Whilst provision of holistic and specialist care via an MDT is yet to be implemented as standardised care for EOT2D across England, certain SCPs have already begun incorporating various components of the approach and found these to be effective. For example, diabetes specialists utilising a person-centred approach in consultations have found this to be extremely effective in engaging PEOT2D.

“… we have to be patient-centred. Because it’s easy to say, yeah, I have my agenda as a healthcare professional, but my patient has their own agenda. So it’s about compromising and agreeing on the fact that you have your agenda. Let’s focus on yours and how we can incorporate the diabetes in your lifestyle. And that is what I have found useful in making them engage with their diabetes.”

(HCP22, Female, 40–59 years)

Similarly, SCPs utilising an MDT approach found this to be effective as it ensured all those involved in the care of the patient were on the same page. Furthermore, the approach provided a useful opportunity to learn from HCPs in other disciplines. However, again, these specialists recognised their limitations in being able to provide holistic care due to the absence of a psychologist.

“We have MDTs as well, not just around medical stuff; we do discuss the psychological impact and what help we can give people. We’d love if we had a psychologist, we’d love a psychologist in our MDT, that would be amazing.”

(HCP11, Female, 40–59 years)

Therefore, it was evident that if provided with the necessary financial, learning and staffing resources, the desired treatment and care for PEOT2D had the potential to be deemed as highly effective by HCPs.

## Discussion

This is the first England-based qualitative study to explore the challenges of living with EOT2D and gaps in T2D care planning from both an individual and HCP perspective. Interpretation of both datasets according to the *Mechanism* constructs of NPT identified the myriad personal and healthcare challenges faced by PEOT2D and suggested the need for future EOT2D treatment and care to involve provision of holistic and specialist care via an MDT. Findings from the HCP data also revealed several barriers and facilitators to implementation of the desired approach to treatment and care within an English healthcare context.

### Challenges of the current treatment and care

Many of the barriers to PEOT2D understanding, engaging, enacting and monitoring their T2D treatment stemmed from GP practices being their main and/or sole caregivers. Views expressed by the young adults living with EOT2D suggested that GP practices struggled to provide the time, expertise, compassion and treatment consistency they desired. As a result, PEOT2D were often left navigating highly fragmented care as they interacted with different HCPs who provided conflicting and misleading information. Our study is not the first to report patient dissatisfaction with T2D care across primary care services. Previous national and international studies have reported similar findings, with people with T2D advocating strongly for improvements in HCP communication skills and overall continuity of GP-delivered care [27, 28]. Despite our efforts to recruit a diverse range of HCPs, we were only able to interview one GP which limited our ability to capture the viewpoints and experiences of PCPs involved in EOT2D treatment and care. Nevertheless, previous research exploring T2D care in England found GP practices to be grappling with extensive pressures that compromised their ability to provide high-quality care, despite their intensions to do so [27]. These findings not only offer a plausible explanation for many of the negative experiences shared by our participants (PETO2D) but also, suggest that shifting caregiving responsibilities to specialists may indeed be a suitable way forward. As well as addressing a major unmet need in EOT2D treatment and care, implementation of this approach could prove beneficial at alleviating some of the existing pressures on GP practices.

### Implementing the desired EOT2D treatment and care

Despite high coherence amongst SCPs regarding the need for a different approach to EOT2D treatment and care, they were also sceptical of successful implementation. Only with provision of relevant funding, learning and staffing (specialists including dietitians and psychologist) resources could specialists develop what they considered the ‘ideal’ MDT for EOT2D. Furthermore, SCPs also recognised that all HCPs across the primary-secondary interface would have to modify how they worked individually and collectively.

Individually, all HCPs across the primary-secondary interface would have to ensure interactions with PEOT2D utilised positive communication and consultation skills including non-judgemental and non-stigmatising behaviours, shared-decision making and personalised care. Despite current NICE (National Institute for Health and Care Excellence) guidance advocating for this exact approach to T2D management [29], our findings suggest that this is yet to become ubiquitous practice across England. Previous studies showed potential for improved self-management behaviours and clinical outcomes amongst people with T2D if they experienced high quality interactions with their HCPs [30, 31]. Therefore, further research exploring HCP barriers, facilitators and motivators to provision of consistent personalised and compassionate care is essential to support long-term implementation of the desired approach to EOT2D treatment.

Collective implementation of the desired approach to EOT2D treatment and care would involve all HCPs across the primary-secondary interface learning to work in a collaborative and coordinated manner. These findings align with previous studies that have highlighted integrated care as the solution to the fragmentation that continues to hinder delivery of high-quality T2D care [32, 33]. Despite offering different solutions to integrated care, it was clear in both studies that the movement of people with T2D between primary and secondary care depending on the complexity of their cases made it difficult to reduce tensions between primary and SCPs. Conflict arose regarding HCP roles, skill level and expectations [32, 33], limiting the HCPs ability to effectively implement integrated care. This perhaps explains why specialists in our study were so keen to oversee care of PEOT2D from the offset. They recognised the daily life demands and personal challenges of PEOT2D in addition to their high levels of risk, and therefore wanted to take proactive measures rather than only seeing these young adults with EOT2D when the severity and complexity of their cases had increased.

Currently 66% of all PEOT2D are receiving care solely from their GP practices [5] and it is evident from our findings that this approach needs modifying. Although other researchers and clinicians have not specified an entire shift in caregiving responsibilities, like our study, they have identified the increasing need to integrate specialist and/or MDT care into the treatment of PEOT2D. The most recent efforts at implementing integrated and improve care for PEOT2D across England comes in the form of the Type 2 Diabetes in the Young (T2Day) programme [34]. This programme will see all Integrated Care Systems (ICSs) across England work together to offer holistic (including psychosocial support) and personalised care to PEOT2D, ensuring optimisation and completion of all clinical care processes in parallel [34]. The vision of the T2Day programme aligns with the desired EOT2D treatment and care identified in our study, and thus holds potential to be a promising step forward if implemented correctly. However, given that implementation will be locally contextualised [34], effectiveness of the programme could vary significantly. HCPs in our study identified a range of strategies pertaining to the many facets of integrated care [35] that could enhance roll out of the T2Day programme across England. Firstly, by recognising the need for provision of holistic and specialist care via an MDT, our approach will help ICSs think about organisational integration and how commissioning and programme delivery can be tailored to this approach. Secondly, HCPs in our study identified the need for a ‘point of contact’ to provide ongoing and synchronous non-clinical support to PEOT2D therefore, allowing ICSs to think about administrative integration. Finally, to ensure successful implementation of the MDT approach, HCPs in our study also highlighted the need for streamlined referrals to specialist services, regular MDT meetings and interprofessional (primary and secondary) team-based learning to ensure delivery of EOT2D care based on shared aims and understanding. Hence, giving ICSs a range of options to promote clinical and service integration.

### Strengths & limitations

This qualitative analysis has many strengths, namely it utilised NPT to interpret data thereby, giving a novel insight into real world implementation strategies for provision of effective EOT2D treatment and care within an English healthcare context. Furthermore, by interviewing both PEOT2D and HCPs, we were able to consider present and future lived experiences from both an individual and HCP perspective. Despite adopting a diverse recruitment pathway, most of our participants were from a white background and female, meaning we were unable to fully explore culture and gender-specific influences on experiences of EOT2D. Further research could consider exploring unmet needs amongst the various subgroups comprising the EOT2D population including ethnic minority groups, LGBTQIA + communities and pregnant women (and those who are planning for pregnancy). The lack of representation from individuals within the 16 to 25 age range also meant we were unable to explore experiences of transition from adolescent to adult care. However, our NPT analysis revealed experiences to be highly similar across those aged 25 to 39 years suggesting there is little age-specific variation amongst this priority population. Nevertheless, this is again something that could be explored in future research. Regarding HCPs, the lack of primary care representation in our study presents a limitation. Recognising that most PEOT2D are solely under GP care, further research into the views and experiences of PCPs (i.e. GPs and practice nurses) is warranted. Finally, although our analysis revealed a clear need to address stigmatising behaviours exhibited by HCPs, we felt NPT was not able to effectively capture the role diabetes-related stigma played in the experiences of PEOT2D for example, through restricted access to diabetes self-monitoring technology and healthcare assumptions about the patient’s diagnosis and lifestyle. However, this is something that we are currently analysing and drafting as a separate manuscript.

### Practice implications

This study has identified the need for provision of holistic and specialist care to PEOT2D. The use of NPT has highlighted barriers and facilitators to implementation within an English healthcare context as well as, implications for emerging EOT2D treatment and care across England. If implemented successfully, findings from our research could help improve the future healthcare experiences (and outcomes) of these high-risk young individuals.

## Conclusions

Findings from this study suggest that GP care alone cannot accommodate the complex needs of PEOT2D. Provision of holistic and specialist care via an MDT is essential to improve the future lived experiences of young adults with EOT2D. The use of NPT within this study has enabled identification of resources barriers, and individual and collective changes required by HCPs across the primary-secondary interface to support implementation. Further research into the views and experiences of PCPs is essential to guide long-term implementation of the desired EOT2D treatment and care.

## Supplementary Information

Supplementary Material 1

Supplementary Material 2

## Data Availability

The data that support the findings of this study are not openly available due to reasons of sensitivity and are available from the corresponding author upon reasonable request. Data are located in controlled access data storage at the University of Leicester.

## Abbreviations

T2D: Type 2 diabetes
EOT2D: Early-onset type 2 diabetes
PEOT2D: People with early-onset type 2 diabetes
RLE: Remaining Life Expectancy
HCPs: Healthcare professionals
UK: United Kingdom
REC: Research Ethics Committee
GP: General practitioner
COM-B: Capability-Opportunity-Motivation-Behaviour Model
NPT: Normalisation Process Theory
HCP: Healthcare professional
SMES: Self-management education and support
GPs: General Practitioners
NIHR: National Institute for Health and Care Research
CRN: Clinical Research Network
SCPs: Specialist Care Providers
MDT: Multidisciplinary Team
PCPs: Primary Care Providers
