# Supplementary material for: Using normalisation process theory to understand implementation of effective early-onset type 2 diabetes treatment and care within England: a qualitative study
Source: BMC Health Serv Res. 2025 Mar 24;25:422. doi: 10.1186/s12913-025-12616-w (PMC11931738; doi:10.1186/s12913-025-12616-w)
Supplement: Supplementary file 1 — Supplementary Material 1 [file 12913_2025_12616_MOESM1_ESM.docx]

**Exploring the unmet needs in adult early-onset type 2 diabetes care (M3)**

**Semi-structured Topic Guide: Adults with early-onset type 2 diabetes (example)**

**Introductory questions**

1. How have you found your experience as a young adult with type 2 diabetes so far

*Prompts*:

- 1. Is there anything that has particularly stood out for you? Whether this was relating to the diagnosis or the treatment you receive? How did this make you feel?

**Resources and Treatment Workload**

1. How confident do you feel managing your diabetes on a day-to-day basis?

*Prompts*:

- 1. For example, how easy/difficult do you find taking your medication, managing and monitoring your glucose levels and making diet and physical activity changes?
  2. For the self-management tasks you find more difficult, can you tell us more about why this is the case?

1. Are there any resources such as information leaflets or books that you have found really useful when it comes to managing your diabetes?

*Prompts*:

- 1. If yes, what were these and how did these help? Were these given to you by your healthcare team? Or did you find them yourself, or through recommendation of family and friends?
  2. What other things do you think could help you manage your diabetes?

**Psychological Resources**

1. Are there any psychological skills that have helped you manage your diabetes? For example, being resilient or self-motivated?

*Prompts*:

- 1. If yes, what were these and how did these help? Did you feel you already had these skills? Or did you healthcare team work with you to help build these skills?
  2. Are there any other psychological skills you feel you need or use to manage your diabetes?

**Barriers to Diabetes Care**

1. Can you tell us a bit about what it’s like arranging appointments with your healthcare team?

*Prompts*:

- 1. Is it easy/hard?
  2. Is there anything you would like to change? For example, the location or timing or format of the appointment.

1. Can you tell us a bit about attending appointments and your experience with your healthcare team?

*Prompts*:

- 1. Is it easy/hard to understand the information you receive during appointments? Is there anything you would change about the information you receive?
  2. Following Covid-19, how has your experience changed when it comes to attending appointments? How did you find attending virtual appointments? If you had to choose between face to face and virtual appointments which would you choose and why?

1. How does the whole consultation make you feel? Prompt: Do you feel it’s equal?

*Prompts*:

1. Do you feel able to voice your thoughts or concerns?
2. What would make the consultation a more positive experience for you?

**Unmet needs and challenges**

1. How have you found balancing your diabetes care alongside other life events? For example, starting a new job, going to university, or starting a family?
2. Did these life events affect your motivation to self-manage your diabetes on a day-to-day basis? For example, when it came to taking your medication, managing and monitoring your glucose levels, or making dietary changes.

*Prompts*:

- 1. If yes, how?
