# Supplementary material for: Using normalisation process theory to understand implementation of effective early-onset type 2 diabetes treatment and care within England: a qualitative study
Source: BMC Health Serv Res. 2025 Mar 24;25:422. doi: 10.1186/s12913-025-12616-w (PMC11931738; doi:10.1186/s12913-025-12616-w)
Supplement: Supplementary file 2 — Supplementary Material 2 [file 12913_2025_12616_MOESM2_ESM.docx]

**Exploring the unmet needs in adult early-onset type 2 diabetes care (M3)**

**Semi-structured Topic Guide: Healthcare Professionals (example)**

**Introductory questions**

1. Can you tell me a bit about your experiences of treating young adults with type 2 diabetes?

*Prompts:*

1. Have you found any significant differences between treating younger adults and older adults?
2. If yes, can you elaborate on these differences?

**Treatment Work/Workload**

1. Can you tell me a bit about your experiences of diabetes self-management behaviours in young adults with type 2 diabetes?

*Prompts:*

1. Are they able to implement certain self-management behaviours, such as taking medication, implementing physical activity or dietary recommendations, more easily than others? Why do think this is the case?

**Resources**

1. As a healthcare professional, do you feel you have adequate training and physical resources to effectively facilitate consultations with young adults with type 2 diabetes? *Elaborate.*
2. What are your thoughts and opinions on the current psychological support available for young adults with type 2 diabetes?

**Barriers and unmet needs in type 2 diabetes care**

1. As a healthcare professional, how do you think physical barriers such as location or time of appointment can impact young adults with type 2 diabetes?

*Prompts:*

1. What do you think can be done to address these physical barriers?
2. Are there any other physical barriers you can think of?
3. From a healthcare professional’s perspective, how do you think social barriers such as language used during consultations and appointments can impact young adults with type 2 diabetes?

*Prompts:*

- 1. What do you think can be done to address these social barriers?
  2. Are there any other social barriers you can think of?

1. How do you think these physical and social barriers can impact motivation for diabetes self-management in a young adult with type 2 diabetes?

*Prompts:*

1. Arranging and taking medication
2. Lifestyle behaviours including diet and physical activity
3. Monitoring/Managing blood glucose
4. How do you feel these barriers impact you as a healthcare professional?

*Prompts:*

- 1. How would you like to see these barriers being addressed?

**Type 2 Diabetes Care Planning**

1. As a healthcare professional, what are your thoughts on the appointment journey for a person with type 2 diabetes?

*Prompts:*

1. Is the journey easy/difficult?
2. How easy/difficult is it to see a young adult with type 2 diabetes?
3. If you had the chance to improve the appointment journey, what would you do differently?
4. What are your thoughts and opinions on the patient information you have for a person with type 2 diabetes?

*Prompts:*

1. Is this sufficient to facilitate a diabetes consultation? Is there any more information that would help? If so, what? How would this help?
2. Is the patient information organised in a way that is easy for you to review and prepare for a consultation? What could be done to make the preparation process easier?

**Unmet needs**

1. What are your thoughts and opinions of the overall type 2 diabetes treatment and care available for young adults with type 2 diabetes?
